# Evaluation of circulating insulin-like growth factor-1, heart-type fatty acid-binding protein, and endotrophin levels as prognostic markers of COVID-19 infection severity

**DOI:** 10.1186/s12985-023-02057-4

**Published:** 2023-05-15

**Authors:** Amal A. Mohamed, Aya A. Nour, Noha M. Mosbah, Alaa S. M. Wahba, Omnia E. Esmail, Basem Eysa, Ahmed Heiba, Hussin H. Samir, Ahmed A. El-Kassas, Ahmed S. Adroase, Ahmed Y. Elamir, Ghada M. Mahmoud, Rasha S. Rafaat, Hatem A. Hassan, Yasmine S. El Abd

**Affiliations:** 1 Biochemistry and Molecular Biology Department, National Hepatology and Tropical Medicine Institute, Cairo, Egypt; 2 Biochemistry and Molecular Biology Department, Faculty of Pharmacy, Suez Canal University, Ismailia, Egypt; 3 Biochemistry and Molecular Biology Department, Faculty of Pharmacy, Egyptian Russian University, Cairo, Egypt; 4 Gastroenterology and Hepatology Department, National Hepatology and Tropical Medicine Institute, Cairo, Egypt; 5 Internal Medicine Department, Medicine and Clinical Studies Research Institute, National Research Centre, Cairo, Egypt; 6 Nephrology Unit, Internal Medicine Department, Faculty of Medicine, Cairo University, Cairo, Egypt; 7 Radiology Department, El-Sahel Teaching Hospital, Cairo, Egypt; 8 Clinical Pathology Department, El-Sahel Teaching Hospital, Cairo, Egypt; 9 Radiology Department, Faculty of Medicine, Cairo University, Cairo, Egypt; 10 Clinical Pathology Department, Faculty of Medicine, Benha University, Benha, Egypt; 11 Neurology and Psychiatry Department, Faculty of Medicine, Minia University, Minia, Egypt; 12 Gastroenterology and Hepatology, Internal Medicine Department, Faculty of Medicine, Minia University, Minia, Egypt; 13 Microbial Biotechnology Department, Biotechnology Research Institute, National Research Centre, Cairo, Egypt

**Keywords:** SARS-CoV-2, IGF-1, HFABP, ETP, COVID-19

## Abstract

**Background:**

Coronavirus Disease 2019 (COVID-19) is a worldwide pandemic challenge spreading enormously within a few months. COVID-19 is characterized by the over-activation of the immune system causing cytokine storm. Insulin-like growth factor-1 (IGF-1) pathway can regulate the immune response via interaction with various implicated cytokines. Heart-type fatty acid-binding protein (H-FABP) has been shown to promote inflammation. Given the fact that coronavirus infections induce cytokines secretion leading to inflammatory lung injury, it has been suggested that H-FABP levels are affected by COVID-19 severity. Moreover, endotrophin (ETP), the cleavage product of collagen VI, may be an indicator of an overactive repair process and fibrosis, considering that viral infection may predispose or exacerbate existing respiratory conditions, including pulmonary fibrosis. This study aims to assess the prognostic capacity of circulating IGF-1, HFABP, and ETP, levels for COVID-19 severity progression in Egyptian patients.

**Methods:**

The study cohort included 107 viral RNA-positive patients and an equivalent number of control individuals with no clinical signs of infection. Clinical assessments included profiling of CBC; serum iron; liver and kidney functions; inflammatory markers. Circulating levels of IGF-1; H-FABP, and ETP were estimated using the corresponding ELISA kits.

**Results:**

No statistical difference in the body mass index was detected between the healthy and control groups, while the mean age of infected patients was significantly higher (*P* = 0.0162) than the control. Patients generally showed elevated levels of inflammatory markers including CRP and ESR concomitant with elevated serum ferritin; D dimer and procalcitonin levels, besides the COVID-19 characteristic lymphopenia and hypoxemia were also frequent. Logistic regression analysis revealed that oxygen saturation; serum IGF-1, and H-FABP can significantly predict the infection progression (*P* < 0.001 each). Both serum IGF-1 and H-FABP as well as O_2_ saturation showed remarkable prognostic potentials in terms of large AUC values, high sensitivity/specificity values, and wide confidence interval. The calculated threshold for severity prognosis was 25.5 ng/mL; 19.5 ng/mL, 94.5, % and for IGF-1, H-FABP, and O_2_ saturation; respectively. The calculated thresholds of serum IGF-1; H-FABP, and O_2_ saturation showed positive and negative value ranges of 79–91% and 72–97%; respectively, with 66–95%, 83–94% sensitivity, and specificity; respectively.

**Conclusion:**

The calculated cut-off values of serum IGF-1 and H-FABP represent a promising non-invasive prognostic tool that would facilitate the risk stratification in COVID-19 patients, and control the morbidity/mortality associated with progressive infection.

## Introduction

Corona Virus Disease 2019 (COVID-19) pandemic emerged in late 2019 caused by the severe acute respiratory syndrome coronavirus 2 (SARS-CoV-2), which belongs to the Coronaviridae family [1] and is mainly transmitted via contact routes and respiratory droplets [2]. COVID-19 is associated with severe respiratory syndrome combined with other systemic complications such as intestinal infections, and renal and heart failure causing high mortality rates. Greater than 243 million positive cases were confirmed worldwide, with more than 6.5 million deaths as of September 28, 2022 [3]. Egypt confirmed its first case of COVID-19 on February 14, 2020, as the first African country with a confirmed case and the first COVID-19-associated mortality by March 8, 2020. The first and second waves of COVID-19 in Egypt were recorded in mid-June and late December 2020, respectively. From February 14th, 2020 to April 9th, 2021, 208,876 laboratory-confirmed infections, including 12,362 deaths (5.92%) by SARS-CoV-2 infection were recorded according to the official website of the Egyptian MOH [4]. Although the incidence and morbidity rates were perceived as low, Egypt was ranked as the 7th country in COVID-19 case fatality rate (CFR 5.92%). The COVID-19 pandemic initiates immunological response resulting in a wide range of disease severities with rapid progression to acute respiratory distress syndrome (ARDS) which is the main cause of death associated with COVID-19 [5]. The suddenly deteriorating conditions of some patients are mainly mediated by uncontrolled activation of the immune response or rapid elevations of inflammatory cytokines leading to a cytokine release syndrome (CRS) [6]. Therefore, suppressing the cytokine storm is important to stop disease deterioration and reduce mortality [7, 8]. Insulin-Like Growth Factor-1 (IGF-1) is a secretory protein produced mainly by the liver and locally in tissues exhibiting autocrine/paracrine activities on cells. IGF1 is the most important mediator of growth hormone (GH) effects [9]. IGF1 has been found to exert pro-survival/anti-aging, anti-inflammatory, and antioxidant with neuro- and hepatoprotective properties [10]. Complex interactions between GH, IGF-1, and the immune system have been confirmed [11]. Available data indicate that the GH/IGF-1 axis exerts both pro-inflammatory and anti-inflammatory effects [12]. Cumulative evidence supports that the IGF-1 pathway can regulate the immune response via interaction with various cytokines and immune cells [13]. It has been shown that higher serum IGF-1 concentrations are associated with a lower COVID-19 severity [14]. Fatty acid-binding proteins (FABP), members of the lipid-binding proteins superfamily, are responsible for the transportation of fatty acids and lipophilic materials into or out of cells [15]. There are several types of tissue-specific FABP, among them FABP-3 which is predominantly distributed in cardiac myocytes and is also named heart-type fatty acid-binding protein (H-FABP) [16]. H-FABP is rapidly released into the circulatory system in response to ischemic injury to the myocardium, then eliminated by the kidney. H-FABP has also been shown to promote inflammation, growth, and migration of vascular smooth muscle cells [17]. Cytokines secreted in response to coronavirus infections lead to inflammatory lung injury, which reduces blood oxygen concentration and puts myocardial cells in a hypoxic state, thereby increasing the release of H-FABP into the blood [18, 19]. Therefore, elevated serum H-FABP can be used as an indicator of severe COVID-19 and an independent risk factor for patient prognosis. Endotrophin (ETP) is a cleavage product of collagen VI (Col VI) that is known to have essential roles in extracellular matrix remodeling. In the lung, Col VI is a component of the basement membrane and provides flexibility and mechanical support [20]. ETP is an adipokine with a major influence on adipose tissue, resulting in the systemic elevation of pro-inflammatory cytokines [21]. It is reported that increased ETP levels were associated with mortality in chronic obstructive pulmonary disease (COPD), which may indicate an over-active repair process and fibrosis [22]. Previous studies indicated that SARS-CoV-infected patients exhibited decreased lung function and increased fibrosis [23, 24]. Several intersecting mechanisms between coronavirus infection and fibrotic pathways have been highlighted that may be targetable to improve patient outcomes [25]. This study aimed to assess the prognostic capacity of circulating IGF-1, HFABP, and ETP levels for COVID-19 infection severity in Egyptian patients during the 3rd wave of the pandemic.

## Subjects and methods

### Subjects

**Fig. 1:**
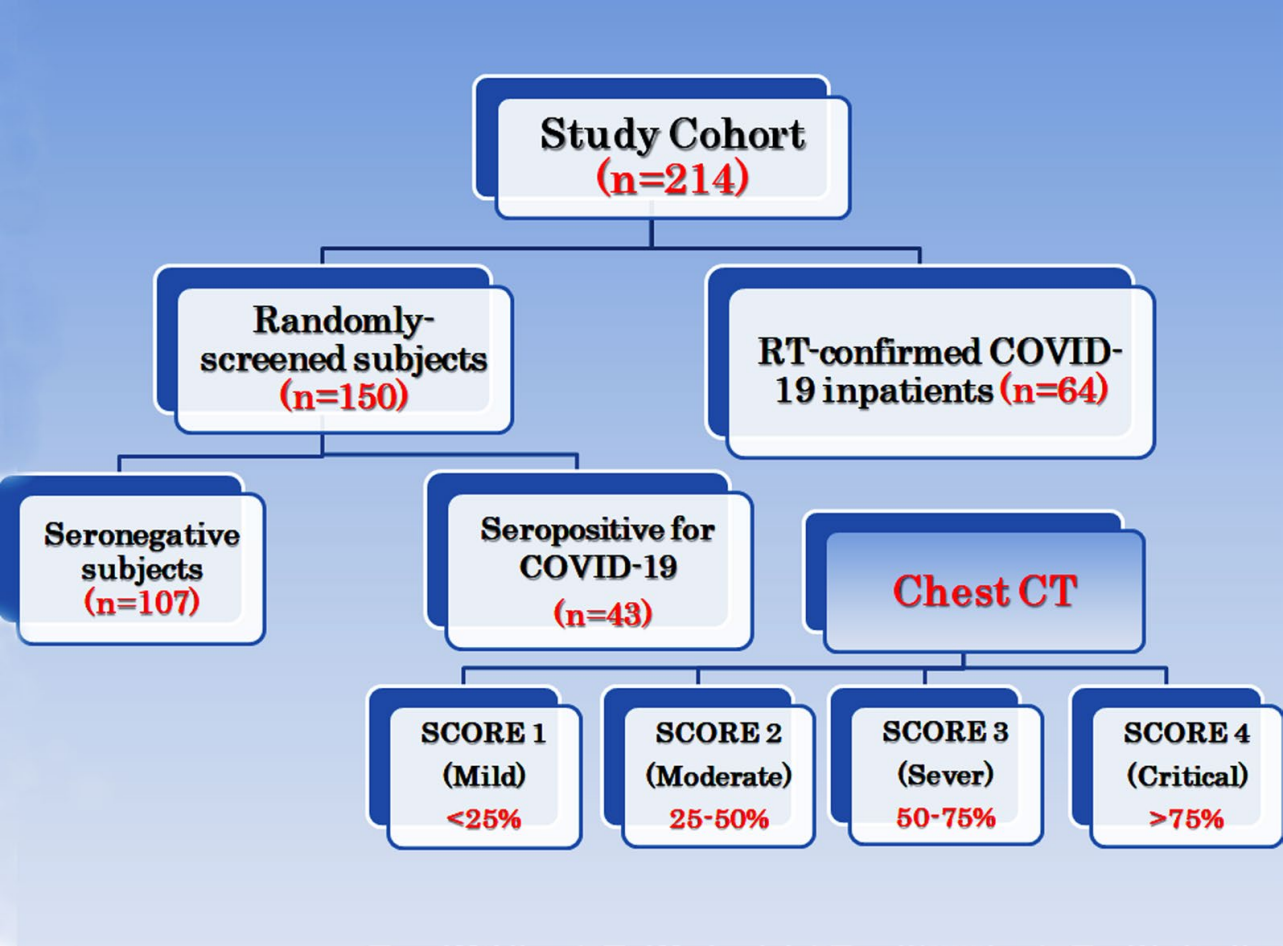
Flow Chart of the study cohort

This study followed STARD 2015 reporting guidelines. Samples were collected during the period of October 2021–July 2022, and the study cohort included a total of 214 subjects categorized as follows: 64 confirmed RT-positive COVID-19 infected inpatients hospitalized at the National Hepatology and Tropical Medicine Research Institute and Ahmed Maher teaching hospital (Cairo-Egypt), and 150 randomly screened subjects attending outpatient clinic using Antibody Test Kit by Labnovation Technologies, China. 107 subjects were COVID-19 IgM/IgG seronegative and were incorporated in the study as negative controls and the remaining 43 subjects were seropositive and further processing was carried out for viral-RNA COVID-19 detection and clinical assessment. All samples were collected during the period of October 2021–July 2022 and thereafter were classified into two equal groups (107/each); negative control with no clinical signs of infection and seronegative for covid test IgM and IgG. Inclusion criteria included age ≥ 21 years; and a positive viral RNA test. Exclusion criteria included patients with bacterial infection and patients with incomplete data. A flow chart of the study cohort is demonstrated in Fig. 1.

### Clinical assessments

Cardiac, and respiratory examinations and full routine laboratory investigations were carried out including CBC, iron profile (Ferritin), cardiac biomarkers D-dimer and lactate dehydrogenase (LDH), blood urea nitrogen, liver enzymes: aspartate aminotransferase (AST), alanine aminotransferase (ALT), inflammation biomarkers: C-reactive protein (CRP), erythrocyte sedimentation rate (ESR), serum procalcitonin, and chest computed tomography (CT). Accurate quantification of circulating levels of IGF-1, H-FABP, and ETP was carried out by Enzyme-Linked Immunosorbent Assay (ELIZA) CAT. NO E1213Hu, E0103Hu, and E7053Hu respectively (Bioassay Technology Lab, Shanghai, China) according to the user manual. The absorbance was measured at 450 nm and the cut-off value considered a positive response was established as readings above the minimum detection limit of each ELISA kit. The overall CT severity score was defined as the sum of points scored in each segment of the twenty lung regions, ranging from 0 to 40 points, with a severity threshold set as 19.5 points [26].

### Statistical analyses

The sample size was calculated using G-Power software version 3.1.9.7 (Fraz faul, Germany). The study has two independent groups. The Priori calculation indicated a difference in the infected group of approximately 2 folds the healthy control group. A minimum of 7 subjects was required to be assigned in each study group to achieve an effect size (f) of 2 and a study power of 95% (1-β error probe). This number was required to reject the null hypothesis that was further evaluated by a continuity-corrected squared Fishers exact test, with a probability of type I error (α error = 0.05), power = 95%. The Data were analyzed using GraphPad Prism 9.5.1. Numerical data was described or mean ± standard deviation as appropriate, while qualitative data were described as numbers and percentages. Chi-square (Fisher's exact) test was used to examine the relation between qualitative variables as appropriate. Multivariate analysis was done by the Logistic regression model to test for the independent predictive effect of statistically significant variables on the univariate level by calculating the odds ratio and its 95% confidence interval. Calculation of sensitivity, specificity, positive predictive value, negative predictive value, and total accuracy with their 95% confidence interval was done using ROC (Receiver operating characteristics) analysis and logistic regression model. Correlation analysis by doing Pearson correlation was done. A *P*-value less than or equal to 0.05 was considered statistically significant. All tests were two-tailed.

## Results

### Baseline demographic and clinical characteristics of the study cohort

The baseline characteristics of the study cohort are summarized in Table 1. Compared to the negative control, the mean age of infected individuals (45.64 ± 9.411, 48.84 ± 12.18; respectively) was observed to be significantly higher (*P* = 0.0162), while no statistical difference was observed in the body mass index (BMI) despite the higher mean value observed in the infected group. The hematological parameters including RBCs; MCH; WBCs, and HCT were significantly lower in infected patients relative to control (*P* = 0.0302, 0.0419, < 0.0001 each; respectively). The typical COVID-19-associated lymphopenia and hypoxemia (low O_2_ saturation) were more frequently observed in the infected cohort (*P* < 0.0001/each), while monocytes, neutrophils, as well as platelets and PT/INR showed higher counts relative to control (*P* = 0.018, < 0.0001 each; respectively). Patients also showed higher levels of liver transaminases; urea; D-dimer (*P* < 0.0001/each); LDH (*P* = 0.0226), and similarly inflammatory markers including CRP; Neutrophils/lymphocytes ratio (NLR); platelets/lymphocytes ratio (PLR); ferritin, and ESR were 3.24, 1.79, 1.86, 2.04, and 1.47 fold higher relative to control; respectively (*P* < 0.0001/each). The levels of IGF-1 and H-FABP were found to be 58.6% lower and 34.74% higher; respectively in patients’ sera (*P* < 0.0001/each), while no statistical difference was observed in ETP levels relative to control. Regarding the frequency of clinical signs of infection, the most common symptoms included dry cough (18.7%); headache (16.8%); nausea (15.9%); abdominal pain (15%); sore throat (12.1%); dizziness and dyspnea (9%); vomiting and diarrhea (8.4 and 7.5%; respectively), and the least frequent symptom was fever in 3.7% of the patient cohort. The frequency of smell/taste loss; unconsciousness, and neurological symptoms (ataxia, acute cerebrovascular disease, seizures, stroke, neuropathy, and delirium) were also higher among COVID-19 patients (*P* < 0.05). Based on chest CT scoring, the infection severity was categorized into mild (score 1); moderate (score 2); severe (score 3), and critical (score 4) was observed in 40.2; 30.8; 15.9, and 13.1% of the patients’ cohort; respectively, with two mortalities recorded; one among moderate and critical cases. To determine the baseline features associated with the high risk of infection severity, a logistic regression analysis was performed using the data of patients with mild (Score 1) infection as a control. Results revealed that both serum IGF-1 and H-FABP, as well as oxygen saturation, were significantly associated with progressive infection severity (Table 2), suggesting that higher levels of serum IGF-1 and H-FABP, and lower oxygen saturation are more likely associated with progressive severity. Notably, mean age showed a significant predictive potential (*P* = 0.003) for moderate severity (score 2), whereas younger age was more likely to have a mild infection.

**Table 1:** Baseline demographic and clinical characteristics of the study cohort (*n* = 214)

	Control (n = 107)	Infected (n = 107)	*P* value
Gender			
Male	62	66	NS*
Female	45	41	
Age (years)	45.64 ± 9.411	48.84 ± 12.18	0.0162
BMI	27.91 ± 4.631	28.47 ± 4.804	NS
Hb (g/dL)	11.37 ± 1.913	11.23 ± 1.498	NS
RBCs (× 106/µL)	3.965 ± 0.4167	3.85 ± 0.4723	0.0302
WBCs (× 103/µL)	7.327 ± 4.41	3.435 ± 1.275	< 0.0001
HCT (%)	42.95 ± 8.651	34.28 ± 3.891	< 0.0001
PLT (× 103/µL)	227.1 ± 57.76	279.7 ± 81.9	< 0.0001
MCV (fL)	67.7 ± 1.987	67.75 ± 3.213	> 0.05
MCH	31.21 ± 2.163	30.55 ± 3.288	0.0419
MCHC	29.93 ± 2.408	30.94 ± 2.522	0.0017
Neutrophils	56.8 ± 6.333	68.91 ± 3.641	< 0.0001
Lymphocytes	36.72 ± 6.832	23.99 ± 4.215	< 0.0001
Monocytes	6.449 ± 2.689	7.224 ± 2.689	0.018
NLR	1.66 ± 0.6436	2.981 ± 0.6414	< 0.0001
PLR	6.432 ± 2.282	12.01 ± 4.053	< 0.0001
Ferritin (ng/mL)	112.5 ± 34.59	230.6 ± 198.3	< 0.0001
D dimer (mg/L)	0.3005 ± 0.2099	1.232 ± 0.535	< 0.0001
Procalcitonin (pg/mL)	255.6 ± 75.92	2639 ± 2717	< 0.0001
IGF-1 (ng/mL)	47.59 ± 14.29	27.89 ± 10.62	< 0.0001
H-FABP (ng/mL)	20.09 ± 9.457	27.07 ± 16.13	< 0.0001
ETP (mg/L)	17.29 ± 4.876	17.39 ± 4.448	NS
CRP (µg/L)	5.146 ± 3.275	16.7 ± 15.35	< 0.0001
ALT (U/L)	23.35 ± 6.535	30.49 ± 6.324	< 0.0001
AST (U/L)	23.25 ± 4.55	33.14 ± 8.823	< 0.0001
Urea (mg/dL)	29.23 ± 8.388	34.77 ± 10.44	< 0.0001
LDH (U/L)	183 ± 32.42	191 ± 25.36	0.0226
ESR-1st h (mm)	11.76 ± 6.451	16.37 ± 12.45	0.0004
ESR-2nd h (mm)	25.67 ± 11.83	37.86 ± 27.66	< 0.0001
Prothrombin time (sec)	13.48 ± 1.354	16.18 ± 2.784	< 0.0001
INR	1.037 ± 0.1041	1.244 ± 0.2142	< 0.0001
O_2_ saturation (%)	97.66 ± 1.748	92.66 ± 4.628	< 0.0001
Consciousness			
Conscious	107 (100%)	88 (82.24%)	< 0.0001
Unconscious	0 (0%)	19 (17.75%)	
Smell/taste			
Yes	107 (100%)	91 (85.04%)	< 0.0001
No	0 (0%)	16 (14.95%)	
Smoking			
Yes	27 (25.23%)	23 (21.49%)	NS
No	80 (74.77%)	84 (78.5%)	
Neurological symptoms			
Yes	1 (0.934%)	106 (99.06%)	0.0352
No	8 (7.47%)	99 (92.52%)	

*HCT* Hematocrit value; *PLT* Platelet count; *MCV* Mean corpuscular volume; *MCH* Mean corpuscular hemoglobin; *MCHC* Mean corpuscular hemoglobin concentration; *ALT* Alanine aminotransferase; *AST* Aspartate aminotransferase; *CRP* C reactive protein; *ESR* Erythrocyte sedimentation rate; *FABP* Fatty acid-binding proteins; *HFABP* Heart-type fatty acid binding protein; *IGF-1* Insulin-like growth factor; *LDH* Lactate dehydrogenase; *NLR* Neutrophils/lymphocytes ratio; *PLR* Platelets/lymphocytes ratio; *INR* International normalized ratio

*NS: Non-significant

**Table 2:** Baseline features predicting infection severity

Severity score	Variable	ß-Coefficient	SE	Wald Chi^2^	*P* value	Odds ratio	95% CI range	
							Lower	Upper
Score 2	Age	− 0.404	0.138	8.554	0.003	0.942	− 0.675	− 0.133
	IGF-1	0.807	0.211	14.616	0.000	1.150	0.393	1.220
	H-FABP	2.049	0.480	18.194	< 0.0001	1.259	1.108	2.991
	O_2_ saturation	− 0.388	0.197	3.856	0.05	0.859	− 0.775	− 0.001
Score 3	IGF-1	1.223	0.282	18.850	< 0.0001	1.236	0.671	1.775
	H-FABP	3.686	0.657	31.455	< 0.0001	1.514	2.398	4.974
	O_2_ saturation	− 0.659	0.210	9.827	0.002	0.772	− 1.07	− 0.247
Score 4	IGF-1	3.207	0.672	22.776	< 0.0001	1.741	1.89	4.525
	H-FABP	4.382	0.735	35.575	< 0.0001	1.637	2.942	5.822
	O_2_ saturation	− 0.745	0.216	11.94	0.001	0.746	− 1.168	− 0.322

### The patterns of predictive variables in patients with different severity scores

**Fig. 2:**
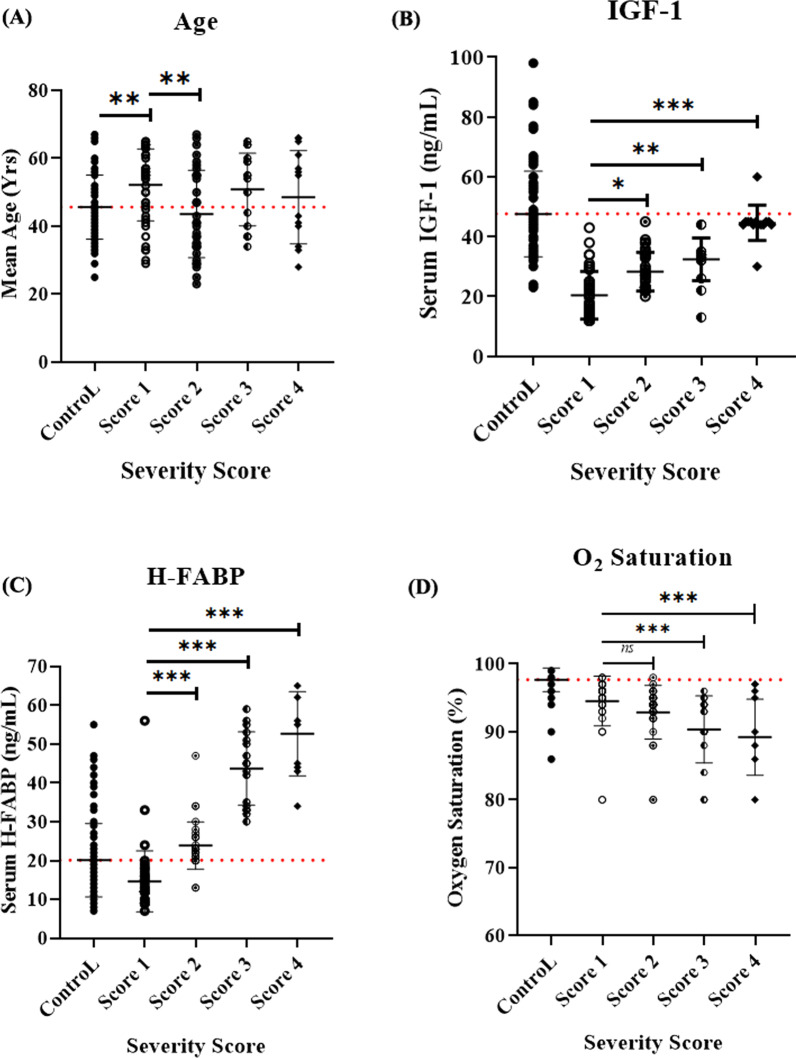
Profile of COVID-19 infection severity predictive variables in patients with different severity scores. No statistical difference was observed in the mean age between patients and the control group except for score 1-patients that had a significantly higher mean age (*P* = 0.0033), while patients with mild infection (score 2) showed lower mean age relative to score 1-patients (*P* = 0.0025) (**A**). Both serum IGF-1 (**B**) and H-FABP (**C**) followed a similar pattern, where they both showed significantly lower levels in score 1-patients compared to the negative control (*P* < 0.0001, 0.0028; respectively), then gradually elevated concomitant with progressive severity score. Patients with all severity scores showed significantly lower oxygen saturation relative to the control group (*P* < 0.0001), with progressive declining percent, observed as the severity score elevates (**D**)

Based on these results, we further assessed the profiles of predictive variables in patients with different severity scores. Compared with the control group, only patients with mild (score 1) infection had a significantly higher mean age (*P* = 0.0033), while patients with moderate (score 2) infection showed lower mean age relative to patients with mild infection (*P* = 0.0025) with no statistical difference observed in other groups compared to both (Fig. 2A). Both IGF-1 and H-FABP followed the same pattern (Fig. 2B and C), where they showed 57.13 and 27.18% lower levels relative to control (*P* < 0.0001, 0.0028; respectively), then their levels progressively elevated concomitantly with infection severity. In contrast, oxygen saturation showed a progressive reduction concomitantly with progressive severity score (Fig. 2D), where patients with all severity scores showed a significantly lower percentage compared to control (*P* < 0.0001).

### Correlation of severity predictive variable with other clinical parameters

**Fig. 3:**
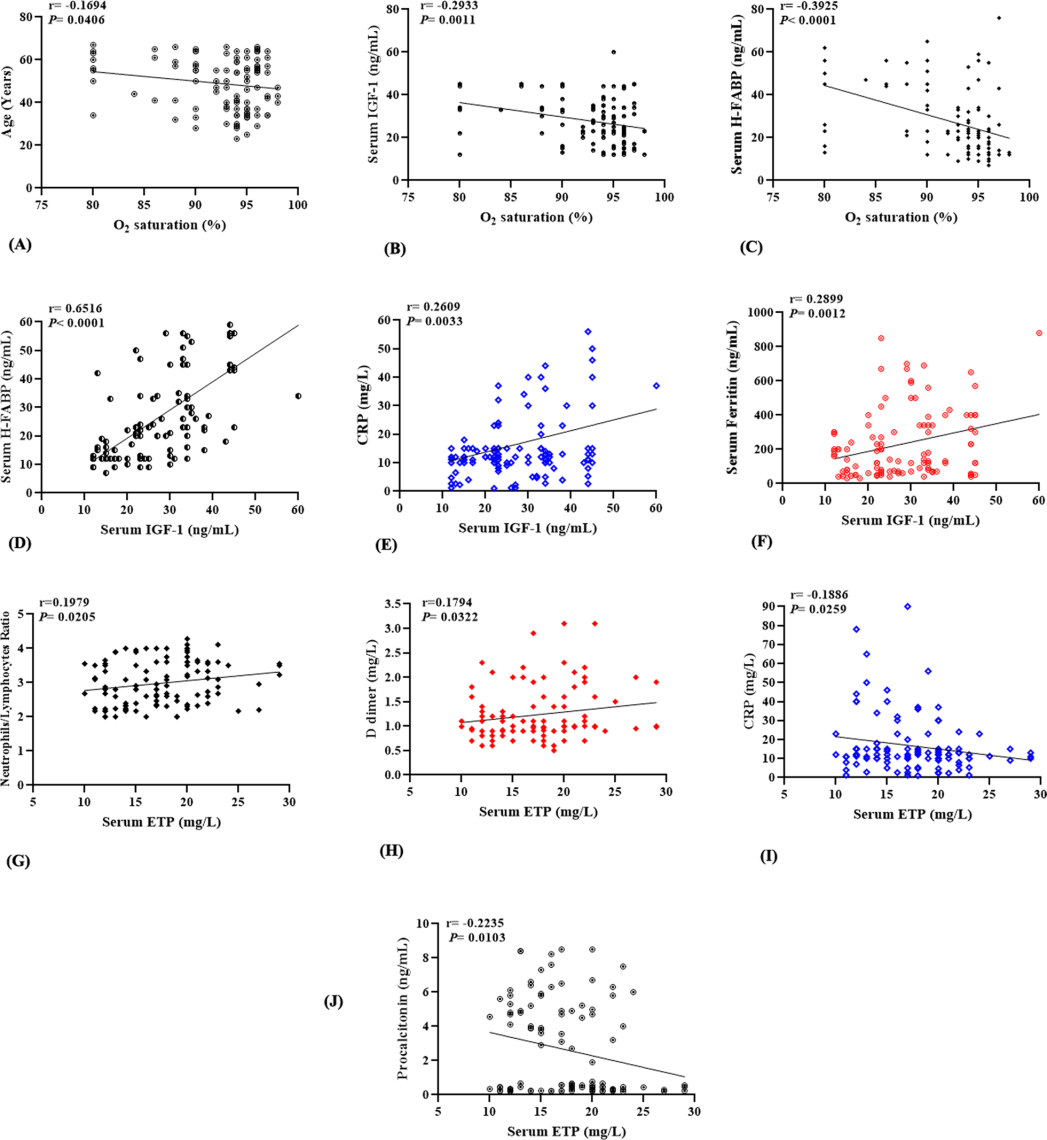
Linear regression of COVID-19 severity predictive variables with other clinical parameters. Oxygen saturation was found to be inversely correlated with age (**A**); IGF-1 (**B**), and H-FABP levels (**C**), while IGF-1 was positively correlated with H-FABP (**D**); CRP (**E**), and ferritin (**F**). Serum ETP also showed positive correlations with NLR (**G**) and D dimer (**H**), and inverse correlations with CRP (**I**) and procalcitonin (**J**)

To explore the factors affecting the dynamics of predictive variables during COVID-19 infection, a multivariate correlation test was performed. Results showed that oxygen saturation was inversely correlated with age (r = − 0.1694,* P* = 0.0406); IGF-1(r = − 0.2933, *P* = 0.0011), and H-FABP (r = − 0.3925, *P* < 0.0001) serum levels (Fig. 3A–C). IGF-1 levels showed a strong positive correlation with H-FABP (r = 0.6516, *P* < 0.0001) (Fig. 3D), while it showed weaker; however significant, correlations with both CRP (r = 0.2609,* P* = 0.0033) (Fig. 3E) and ferritin (r = 0.2899, *P* = 0.0012) (Fig. 3F). Despite the absence of significant difference of serum ETP levels between control and patients’ groups, they showed positive correlations with both NLR (r = 0.1979,* P* = 0.0205) (Fig. 3G) and D dimer (r = 0.1794,* P* = 0.0322) (Fig. 3H), while inversely correlated with CRP (r = − 0.1886,* P* = 0.0259) (Fig. 3I) and procalcitonin (r = − 0.2235,* P* = 0.0103) (Fig. 3J).

## Determination of prognostic threshold for COVID-19 infection severity

**Fig. 4:**
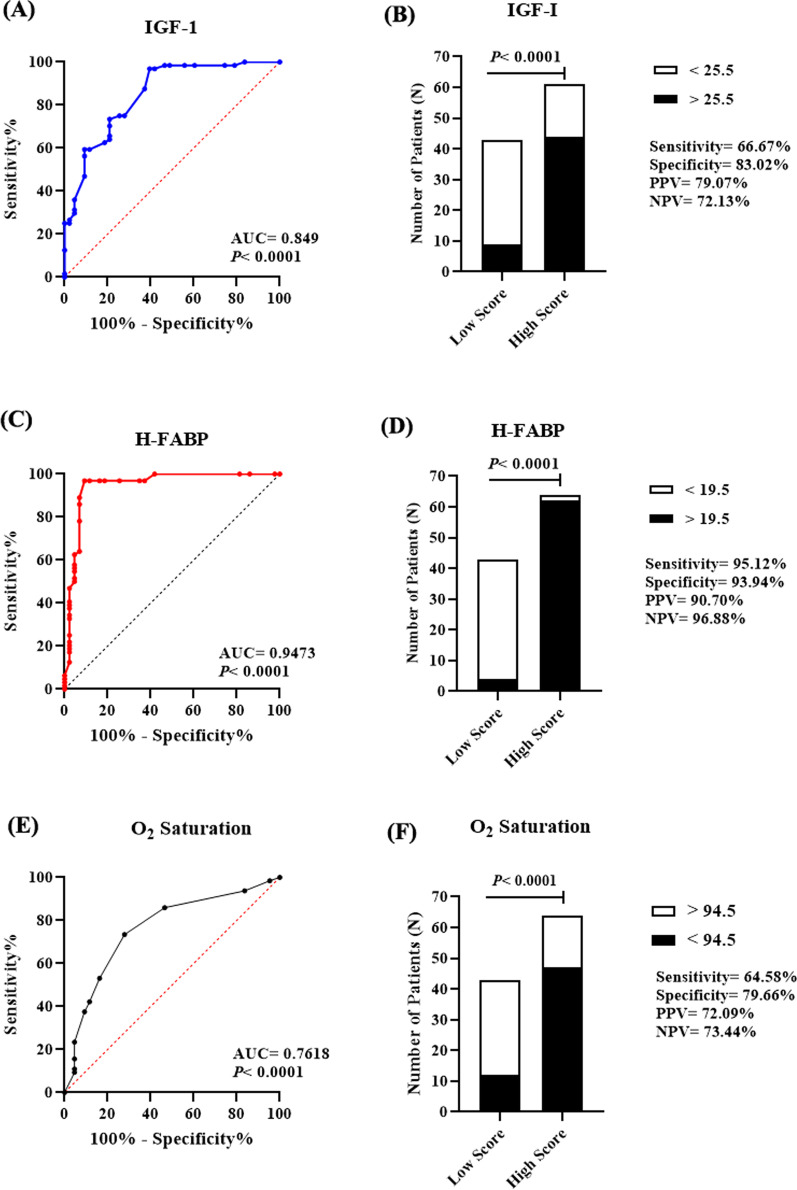
Determination of severity predictive thresholds. Results of ROC curve analysis of serum IGF-1 (**A**); H-FABP (**C**), and O_2_ saturation (**E**) revealed AUC values of 0.849, 0.9473, and 0.7618; respectively (*P* < 0.0001 each). The calculated thresholds of serum IGF-1 (**B**); H-FABP (**D**), and O_2_ saturation (**F**) showed a remarkable prognostic capacity for COVID-19 infection severity, with sensitivity and specificity ranges of 66–95%, 78–94%; respectively

To determine the ideal threshold for COVID-19 infection severity, the selected predictive variables were assessed for the significant area under the curve (AUC) using the receiver operating characteristic (ROC) curve test, using the values of both markers in patients with the least severity score (score 1) as control. Results indicated the remarkable prognostic potential of both serum IGF-1 (Fig. 4A) and H-FABP (Fig. 4C), as well as O_2_ saturation (Fig. 4E), demonstrated as large AUC values with considerable sensitivity and specificity, and wide confidence interval. Meanwhile, analyzing age did not produce an AUC with enough predictive potential (0.6177), and thusly age variable was excluded. The prognostic capacity of the calculated cut-off values of IGF-1; H-FABP, and O_2_ saturation were further validated as revealed by Fisher’s exact test results, showing positive and negative predictive values that ranged between 79–91% and 72–97%; respectively, with 66–95%, 83–94% sensitivity and specificity; respectively (Fig. 4B, D and F). AUC values and the calculated thresholds with their positive/negative predictive values are listed in Table 3.

**Table 3:** Assessment of prognostic thresholds of serum IGF-1, H-FABP, and O_2_ saturation

TEST	IGF-1	H-FABP	O_2_ saturation
*ROC curve*			
AUC	0.849	0.9473	0.7618
St. error	0.03811	0.02612	0.04817
95% CI	0.7743–0.9237	0.8961–0.9985	0.6674–0.8562
*P*-value	*P* < 0.0001		
Prognostic threshold	> 25.5 ng/mL	> 19.5 ng/mL	< 94.5%
Sensitivity	73.44% (95% CI 61.52–82.70%)	96.88% (95% CI 89.30–99.44%)	73.44% (95% CI 61.52–82.70%)
Specificity	79.07% (95% CI 64.79–88.58%)	90.7% (95% CI 78.40–96.32%)	72.09% (95% CI 57.31–83.25%)
*Fisher’s exact test*			
PPV	79.07% (95% CI 0.6479–0.8858)	90.70% (95% CI 0.7840–0.9632)	72.09% (95% CI 0.5731–0.8325)
NPV	72.13% (95% CI 0.5983–0.8181)	96.88% (95% CI 0.8930–0.9944)	73.44% (95% CI 0.6152–0.8270)
Sensitivity	66.67% (95% CI 0.5297–0.7803)	95.12% (95% CI 0.8386–0.9913)	64.58% (95% CI 0.5044–0.7657)
Specificity	83.02% (95% CI 0.7077–0.9080)	93.94% (95% CI 0.8543–0.9762)	79.66% (95% CI 0.6773–0.8796)
Relative risk	2.837 (95% CI 1.883–4.443)	29.02 (95% CI 8.445–105.6)	2.714 (95% CI 1.764–4.302)
Odds ratio	9.778 (95% CI 3.970–23.29)	302.3 (95% CI 48.56–1386)	7.142 (95% CI 2.9–16.76)

## Discussion

No doubt that gaining insights into SARS-CoV-2 infection progression is vital for proper risk assessment. This approach though is challenged by the fact that most asymptomatic cases or those with mild symptoms are not often hospitalized, which eventually impacts the risk assessment approach. In this report, we meant to carefully select the subjects of the study cohort so that it would cover all of the documented severity scores observed during the successive waves of SARS-CoV-2. The main objective was to investigate clinical biomarkers that would predict the progression of infection severity, with a special focus on circulating IGF-1; H-FABP, and ETP.

Overall, the clinical assessment of infected patients revealed that the most common symptoms reported included dry cough, headache, gastrointestinal symptoms (nausea & abdominal pain), and sore throat, with lower frequencies of dizziness, dyspnea, vomiting, and diarrhea. Strikingly, the least frequent symptom reported was fever (only 3.7%), which conformed to Omicron-associated changing symptoms of SARS-CoV-2 infection characterized with low-grade/or no fever compared with the wild type and other variants [27, 28]. Besides the hypoxemia, the infected patients also showed an altered hematological profile that included lymphopenia, lower counts/estimates of RBCs; MCH; WBCs, and HCT, and higher counts of monocytes, neutrophils, platelets, and PT/INR. In addition, altered cardiac and hepatic functions as well as upregulated inflammatory markers were also reported in agreement with the typical COVID-19-associated hematological abnormalities [29, 30].

Despite the significant difference in ESR observed in infected patients compared to the control, only 53.27% showed ESR values > 25 mm/h, with less than 20% showing values > 50 mm/h. However, according to what was recently reported by Kurt et al. [31], ESR is not prognostic for SARS-CoV-2 infection severity, with lower sensitivity and specificity values for pneumonia, intensive care requirements, and mortality compared with CRP. Notably, the mean age of infected patients was observed to be significantly higher compared to healthy individuals, which was later demonstrated to have a significant prediction of mild-to-moderate infection severity transition that was absent in the higher severity score. Although this finding contradicts the reportedly higher susceptibility of elders relative to developing severe infection observed during the early waves of wild-type COVID-19 [32], our study coincided with the circulation of SARS-coV-2 variants B.1.617.2 “delta” and Omicron; both associated with a high risk of acute infection among unvaccinated adolescents and children [33].

The impact of the pleiotropic growth factor IGF-I on lung pathology has been controversial, where elevated IGF-1 levels were reported in the broncho-alveolar lavage fluid of early ARDS patients [34], whereas low circulating levels were recently proposed as a potential predictor of SARS-CoV-2 infection severity and mortality [14, 35–37]. Results of the current work, however; demonstrated that low levels of circulating IGF1 in patients with mild infection (score 1) escalate concomitantly with progressive infection scores, reaching the maximum in critical cases (score 4). This profile mimics bronchopulmonary dysplasia in which pulmonary levels of IGF1 are upregulated while the circulating levels decline [38] and suggest that low levels of circulating IGF-1 might be merely markers of protein/muscle degradation, particularly that they were inversely correlated with oxygen saturation in one hand, and positively correlated with CRP levels in the other, in contrast to previous reports [39–41]. This hypothesis was further supported by the results of logistic regression that demonstrated IGF-1 predictive power of mild-to-severe infection transition, in terms of higher values of β-coefficient and significance with each higher severity score.

Similarly, the myocardial injury marker H-FABP showed a remarkable predictive power for severity progression, where serum levels in patients were consistently elevated with progressive infection severity scores, which agrees with the recent study of Yin et al. [42] proposing serum H-FABP as a predictive biomarker for COVID-19 severity. Interestingly, serum H-FABP followed the same pattern and further showed a strong positive correlation with serum IGF-1, while inversely correlated with oxygen saturation. This finding implies that IGF-1 might be considered an inflammatory mediator implicated in SARS-CoV-2-associated damage of the pulmonary gas exchange barrier, leading to hypoxia that would prompt ischemia or heart failure [43]; or even directly exerts its inflammatory impact on the heart muscle causing myocarditis demonstrated as elevated serum H-FABP levels. This hypothesis was supported by the positive correlation observed between IGF-1 and H-FABP, CRP, and ferritin levels on one hand, and the inverse correlation between both IGF-1 and H-FABP with O_2_ saturation on the other. Furthermore, our results agreed with the previous findings of Li et al. [44] who reported the inflammatory role of IGF-1 in aggravating ALI during H1N1 infection through PI3K/AKT and MAPK signaling pathways.

In line with these results, the oxygen saturation also showed predictive potentials for infection severity, which is not surprising given that marked hypoxemia is the main prominent feature of SARS-CoV-2 lung pathology [45]. The inverse correlation found between O_2_ saturation and patients’ age in this study agreed with the findings of Sirohiya et al. [46] who reported the inverse association between age and the PF ratio (the ratio of arterial oxygen partial pressure/fractional inspired oxygen received). Regarding endotrophin (ETP), it showed a positive correlation with NLR recently identified as an early marker of critical COVID-19 infection [47] despite the absence of statistical difference in serum levels between patients and controls, which might reflect a subclinical chronic obstructive pulmonary disease (COPD) that often progress to post-COVID pulmonary fibrosis, particularly in severe infection cases [48]. However, investigating this hypothesis requires further follow-up of the recovered patients which was beyond the scope of the current observational study. The prognostic cut-off values of circulating IGF-1; H-FABP, and O_2_ saturation calculated by the receiver operating characteristic (ROC) curve showed an overall large area under the curve (AUC) for the three markers, with remarkable sensitivity and specificity values and a considerable 95% confidence interval range. Moreover, the calculated thresholds showed considerable predictive capacity, with positive and negative predictive values ranging between 79–91% and 72–97%; respectively, 66–95%, and 83–94% sensitivity and specificity; respectively. These results highlight the proposed biomarkers as promising tools for risk stratification/assessment of SARS-CoV-2 infection severity.

The main strong point of this study lies in the new perception of IGF-1 as an inflammatory mediator that has an overt contribution in the SARS-Cov-2 infection progression, which might consolidate the reportedly Omicron-associated changing clinical profile of the disease. However, the sample size is one of the limiting factors that limited the validation of these findings. Therefore, future studies with larger sample sizes are required, particularly with the emergence of new Omicron lineages XBB and XBB-1.5 recently reported.

## Conclusion

While most studies approaching the risk assessment of SARS-CoV-2 infection progression usually focus on severe/critical case prognosis, to the best of our knowledge; this is the first research aiming to determine the earliest clinical markers that predict the infection severity determined in merely mild infection, providing better risk stratification. The proposed cut-off values represent a promising laboratory index for assessing the outcome of patients with COVID-19 and assessing the risk of infection progression in association with the clinical context.

## Data Availability

Inquiries about data availability should be directed to the corresponding author on reasonable request due to the privacy of patients’ results.

## Abbreviations

ALI: Acute lung injury
ALT: Alanine aminotransferase
ARDS: Acute respiratory distress syndrome
AST: Aspartate aminotransferase
AUC: Area under the curve
CBC: Complete blood count
COPD: Chronic obstructive pulmonary disease
COVID-19: Corona virus disease 2019
CRP: C reactive protein
CRS: Cytokine release syndrome
CT: Chest computed tomography
ELISA: Enzyme-linked immunosorbent assay
ESR: Erythrocyte sedimentation rate
FABP: Fatty acid-binding proteins
GH: Growth hormone
HFABP: Heart-type fatty acid binding protein
IGF-1: Insulin-like growth factor
LDH: Lactate dehydrogenase
NLR: Neutrophils/lymphocytes ratio
PLR: Platelets/lymphocytes ratio
ROC: Receiver operating characteristic
SARS-CoV-2: Severe acute respiratory syndrome coronavirus 2
